# Supplementary material for: Binary outcomes of enhancer activity underlie stable random monoallelic expression
Source: eLife. 2022 May 26;11:e74204. doi: 10.7554/eLife.74204 (PMC9135403; doi:10.7554/eLife.74204)
Supplement: Supplementary file 1. — Guide RNAs (sgRNAs) used to generate germline enhancer deletion mice via electroporation or microinjection are displayed. A flanking guide pair was used to delete the indicated enhancer, except for in the case of Klrk15′E, where two sets of flanking guides were used (all four sgRNAs were simultaneously delivered to embryos). Primers used to genotype mice carrying a deletion allele and mice lacking a WT allele are also shown. These primers allow delineation of WT, heterozygous and homozygous enhancer deletion animals with respect to the indicated enhancer element. More than one primer is shown if PCR was performed as a nested reaction; “1” indicates use in the first amplification and “2” indicates use in the subsequent amplification. [file elife-74204-supp1.docx]

| **Target enhancer** | **sgRNA sequence 5’ 🡪 3’** | | **Deletion genotyping primers 5’ 🡪 3’** | | | **Homozygosity genotyping primers 5’ 🡪 3’** | |
| --- | --- | --- | --- | --- | --- | --- | --- |
|  | **Upstream sgRNA** | **Downstream sgRNA** | **Upstream** | **Downstream** | | **Upstream** | **Downstream** |
| ***Klra1_Hss1_*** | CTTAGTGCTTGAGCCCATGA | CAGCATAATACAGGAGGTAA | AAGGCACATACCACATTGTCAC | | GAGCAGTACCTTCCTCTAAGTTC | GTCCAAGGGTGTGACTGGAAG | GAGCAGTACCTTCCTCTAAGTTC |
| ***Klrc1_5_*_′_*_E_*** | CAGGATAATTATTATGATTG | GAGGCACCGTTCAGATGCAG | 1-ATGAGTGTGCAGTGGTGTCTTC  2-TGTGCCAGCCATAAGAGTTTG | | 1-TCATCCAAAGAGCCACAGCA  2-TCCAGATGATGGCTAACTCTCCAT | AGAGACACTTTGTACCTTCCAC | TGTGTCATTGAAGGTTGAACAG |
| ***Klrk1_5_*_′_*_E_*** | ATAGCCAACATTATACTAGA  CTTTCAACTATTATTTAACA | AGCACAAGGTGAGTCCTAGG  TTCTACAACCATTATGTGGG | 1-AAGGAAACCAGAACCCTGATG  2-AGAGTGATCTCAGTGATGCAAGGA | | 1/2-AGCTGAGGACAAGCTGCACA | CATCCATTCAA CATAGTTCTGG | TAACTGATGGTTTTCTTGGCCA |
| ***Klra7_Hss5_*** | AGAACAGTCATTTCTTTAAA | AGAGGGATTACTCTGGGGAA | 1-AGCTTCTGCCTATACTCCTGATTG  2-AAACTTGGGAAAGAGATTCGGAC | | 1/2-AAGGACAACTTTGATGGGTTATGG | TTTTCGCTCATGTCTACCCAG | GGGAGAAGTAGCAGCAGTGT |

Supplementary File 1
