# Supplementary material for: Binary outcomes of enhancer activity underlie stable random monoallelic expression
Source: eLife. 2022 May 26;11:e74204. doi: 10.7554/eLife.74204 (PMC9135403; doi:10.7554/eLife.74204)
Supplement: Supplementary file 2. — Guide RNAs (sgRNAs) used in the ex vivo NK cell enhancer deletion assay are displayed. Non-targeting sgRNA pairs 1 (nt1) and 2 (nt2) were used as negative controls in Figure 3—figure supplement 1A. Primers used to detect the presence of the intended deletion in nucleofected NK cells using the indicated sgRNAs used are also shown. More than one primer is shown if PCR was performed as a nested reaction; “1” indicates use in the first amplification and “2” indicates use in the second amplification. [file elife-74204-supp2.docx]

|  |  | |  | |
| --- | --- | --- | --- | --- |
| **sgRNA target** | **sgRNA sequence 5’ 🡪 3’** | | **Deletion detection primers 5’ 🡪 3’** | |
|  | **Upstream** | **Downstream** | **Upstream** | **Downstream** |
| ***Klrc1_5_*_′_*_E_*** | CAGGATAATTATTATGATTG | GAGGCACCGTTCAGATGCAG | 1-ATGAGTGTGCAGTGGTGTCTTC  2-TGTGCCAGCCATAAGAGTTTG | 1-TCATCCAAAGAGCCACAGCA  2-TCCAGATGATGGCTAACTCTCCAT |
| ***Non-targeting pair 1 (nt1)*** | CAGTCAGGACAGGAACAAGC | CCTCACAGAAACATACATAA | N/A | N/A |
| ***Non-targeting pair 2 (nt2)*** | AGCTGATTCAAGACCAGCCA | CCTTTATGTATGTTTCTGTG | N/A | N/A |
| ***Cd45 (Ptprc)*** | GAGCCTACCAATAGTGCTG | N/A | N/A | N/A |
| ***Klra7_Hss1_*** | GGGCTCAAGCACTCAGAGCA | ACCAAAGTACAGCATAATAT | 1-CTGTGCATGTTGTCAATACAGTG  2-GTAGTTAGTGTCTGTTGGTTAG | 1/2-CAGAATGGGCTTCTTTGTTGGTT |

Supplementary File 2
