# Supplementary material for: Binary outcomes of enhancer activity underlie stable random monoallelic expression
Source: eLife. 2022 May 26;11:e74204. doi: 10.7554/eLife.74204 (PMC9135403; doi:10.7554/eLife.74204)
Supplement: Supplementary file 3. — Intron-spanning primers detecting a region of the Ptprc transcript containing 3 allele-informative SNPs. [file elife-74204-supp3.docx]

|  |  | |
| --- | --- | --- |
| **sgRNA target** | **sgRNA sequence 5’ 🡪 3’** | |
|  | **Upstream** | **Downstream** |
| ***Ptprc/Cd45*** | GCAGACAGATTTGGGGATTCCA | CCTTGTCCGGACGATCTGCT |

Supplementary File 3
