# Supplementary material for: Binary outcomes of enhancer activity underlie stable random monoallelic expression
Source: eLife. 2022 May 26;11:e74204. doi: 10.7554/eLife.74204 (PMC9135403; doi:10.7554/eLife.74204)
Supplement: Source data 2. — Original agarose gel images corresponding to Figure 1—figure supplement 2 E and H are provided in both annotated and unannotated format. The relevant lanes are highlighted and labeled. The cropped lanes are denoted with a white vertical bar. [file elife-74204-data2.zip › Original gel images/Figure1--figure supplement 2 E annotated.pdf]

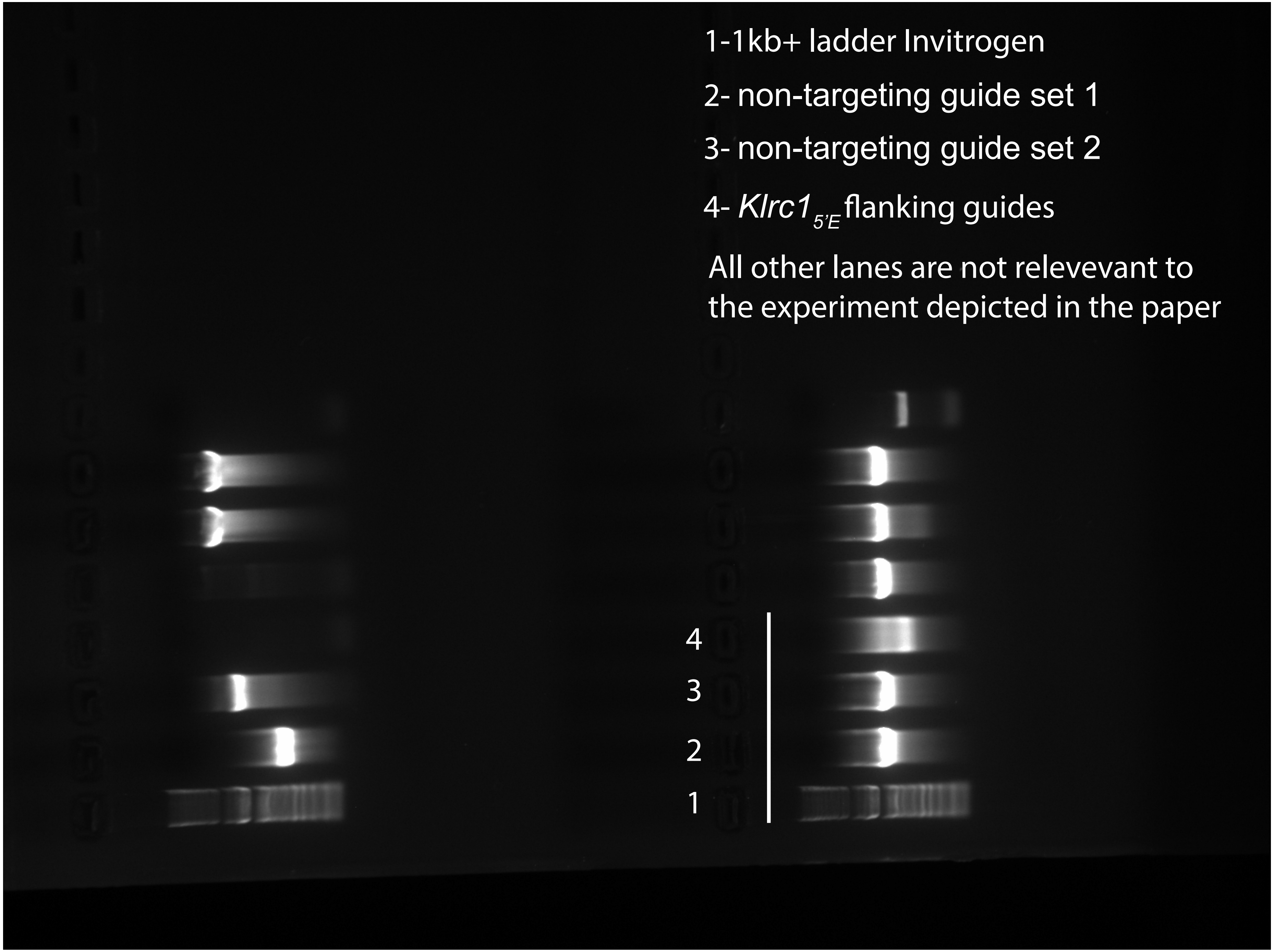
1-1kb+ ladder Invitrogen
2- non-targeting guide set 1
3- non-targeting guide set 2
4- *Klrc1*$_{5'E}$ flanking guides
All other lanes are not relevevant to the experiment depicted in the paper
4
3
2
1
