# Supplementary material for: Binary outcomes of enhancer activity underlie stable random monoallelic expression
Source: eLife. 2022 May 26;11:e74204. doi: 10.7554/eLife.74204 (PMC9135403; doi:10.7554/eLife.74204)
Supplement: Transparent reporting form [file elife-74204-transrepform1.docx]

***eLife’s* transparent reporting form**

We encourage authors to provide detailed information *within their submission* to facilitate the interpretation and replication of experiments. Authors can upload supporting documentation to indicate the use of appropriate reporting guidelines for health-related research (see [EQUATOR Network](http://www.equator-network.org/%20)), life science research (see the [BioSharing Information Resource](https://biosharing.org/)), or the [ARRIVE guidelines](http://www.plosbiology.org/article/info:doi/10.1371/journal.pbio.1000412) for reporting work involving animal research. Where applicable, authors should refer to any relevant reporting standards documents in this form.

If you have any questions, please consult our Journal Policies and/or contact us: [editorial@elifesciences.org](mailto:editorial@elifesciences.org).

**Sample-size estimation**

- You should state whether an appropriate sample size was computed when the study was being designed
- You should state the statistical method of sample size computation and any required assumptions
- If no explicit power analysis was used, you should describe how you decided what sample (replicate) size (number) to use

Please outline where this information can be found within the submission (e.g., sections or figure legends), or explain why this information doesn’t apply to your submission:

We did not perform formal power analyses, but group sizes were based on our experience from previous studies with variability in the measurements performed. Where relevant, sample sizes, statistical methods and number of experimental replicates are indicated in the figure legends. The overall statistical approaches used depended on the number of groups being compared and are described in the M&Ms. In vivo genetic experiments using CRISPR/Cas9-edited mice were performed with group sizes determined by segregation of alleles for 3 genetic configurations: homozygous WT, heterozygous WT/Deletion, or homozygous Deletion/Deletion; group sizes in almost all cases were at least n=3 mice. In cases where a group size was <3, repeat experiments were carried out in which those group sizes were ≥3, with similar results

**Replicates**

- You should report how often each experiment was performed
- You should include a definition of biological versus technical replication
- The data obtained should be provided and sufficient information should be provided to indicate the number of independent biological and/or technical replicates
- If you encountered any outliers, you should describe how these were handled
- Criteria for exclusion/inclusion of data should be clearly stated
- High-throughput sequence data should be uploaded before submission, with a private link for reviewers provided (these are available from both GEO and ArrayExpress)

Please outline where this information can be found within the submission (e.g., sections or figure legends), or explain why this information doesn’t apply to your submission:

The numbers of experimental replicates performed are indicated in the figure legends. In general, at least 2 independent biological experiments were performed for *in vivo* genetic analysis of enhancer function, and in all cases the two or more experiments yielded extremely similar results. In most cases, 2 independently isolated alleles were analyzed for each enhancer deletion. Ex vivo NK cell editing experiments were performed with 3 biological replicates per targeted locus. Assessment of allelic expression of non-NK receptor genes was performed with at least 2 biological replicates. Sorted sequencing experiments (ATAC-seq and CUT&RUN) were generally performed using cells sorted from a single F_1_ hybrid animal according to receptor expression states and used to analyze a single locus. These results were in all cases cross-validating (e.g. histone modification data from CUT&RUN mirrored the accessibility results from ATAC-seq). Furthermore, various histone modifications were cross-validating of each other with respect to both active and repressive modifications.

**Statistical reporting**

- Statistical analysis methods should be described and justified
- Raw data should be presented in figures whenever informative to do so (typically when N per group is less than 10)
- For each experiment, you should identify the statistical tests used, exact values of N, definitions of center, methods of multiple test correction, and dispersion and precision measures (e.g., mean, median, SD, SEM, confidence intervals; and, for the major substantive results, a measure of effect size (e.g., Pearson's r, Cohen's d)
- Report exact p-values wherever possible alongside the summary statistics and 95% confidence intervals. These should be reported for all key questions and not only when the p-value is less than 0.05.

Please outline where this information can be found within the submission (e.g., sections or figure legends), or explain why this information doesn’t apply to your submission:

Statistical analyses, n, and P values are provided in the figure legends. Statistics are further described in the M&M. The results of all statistical tests are provided with raw values in Source Data 2.

(For large datasets, or papers with a very large number of statistical tests, you may upload a single table file with tests, Ns, etc., with reference to sections in the manuscript.)

**Group allocation**

- Indicate how samples were allocated into experimental groups (in the case of clinical studies, please specify allocation to treatment method); if randomization was used, please also state if restricted randomization was applied
- Indicate if masking was used during group allocation, data collection and/or data analysis

Please outline where this information can be found within the submission (e.g., sections or figure legends), or explain why this information doesn’t apply to your submission:

In most cases we compared segregants from genetic crosses based on genotype to compare, such that allocation to group sizes was random. In cases where groups of different genotypes were allocated before comparison, group sizes were allocated equally.

**Additional data files (“source data”)**

- We encourage you to upload relevant additional data files, such as numerical data that are represented as a graph in a figure, or as a summary table
- Where provided, these should be in the most useful format, and they can be uploaded as “Source data” files linked to a main figure or table
- Include model definition files including the full list of parameters used
- Include code used for data analysis (e.g., R, MatLab)
- Avoid stating that data files are “available upon request”

Please indicate the figures or tables for which source data files have been provided:

Source Data 1 provides the original unedited gel images for Figure 1—figure supplement 2. Source Data 2 provides all raw data that were quantified graphically. (Fig. 1-- supplement 2 D and G, Fig. 3 C and F, Fig. 3-- supplement 1B, Fig. 3--supplement 1D, Fig. 3--supplement 2C, Fig. 3--supplement 2F, Fig. 4, Fig. 4-- supplement 1B, Fig. 5, Fig. 5-- supplement 1 B and C, Fig. 7B, Fig. 7-- supplement 1, Fig. 7-- supplement 2H
